# Emergent laparoscopic dome resection and omental suturing to the splenic parenchymal edge for a spontaneously ruptured non-parasitic large splenic cyst in a pediatric patient: a case report

**DOI:** 10.1186/s40792-019-0750-2

**Published:** 2019-12-18

**Authors:** Kumiko Shono, Yoshiko Hashimoto, Takeshi Shono

**Affiliations:** Department of Pediatric Surgery, National Hospital Organization, Kokura Medical Center, Kitakyushu, 803-8533 Japan

**Keywords:** Ruptured splenic cyst, Laparoscopy, Dome resection

## Abstract

**Background:**

Spontaneously ruptured large splenic cyst is a rare in children, and traditionally total or partial splenectomy has been performed for treating an emergent case. We herein present a first case with spontaneously ruptured pediatric splenic cyst treated with emergent laparoscopic dome resection with omental suturing to the parenchymal edge of the spleen.

**Case presentation:**

A 12-year-old girl with a spontaneously ruptured large non-parasitic splenic cyst (SC) was successfully treated by emergent laparoscopic dome resection with omental suturing to the edge of the splenic parenchyma. The patient presented with acute abdominal pain and was diagnosed with a ruptured non-parasitic SC and peritonitis by contrast-enhanced computed tomography (CT). Emergent laparoscopic dome resection of the SC and omental suturing to the splenic parenchymal edge were then performed. The protruding part of the cyst wall was completely resected using an ultrasonically activated device (USAD), and the greater omentum was then sutured to the anterior edge of the splenic parenchyma under a laparoscopic view. No complications were observed during the operation. A histological examination revealed a congenital splenic cyst lined by epithelial cells. The postoperative course was uneventful, and an ultrasound scan showed no evidence of cyst recurrence at 3 years after the operation.

**Conclusions:**

This minimally invasive laparoscopic procedure was feasible and effective for treating a ruptured large splenic cyst in an emergent pediatric patient.

## Background

Congenital splenic cysts (SCs) are uncommon [1], and spontaneously ruptured SCs are extremely rare in children, with few cases reported in the English literature [2, 3]. Although non-parasitic SCs have been managed currently by conservative treatment or spleen-preserving surgery [1, 4], ruptured SCs have traditionally been treated by urgent total splenectomy [2, 5]. We herein report the case of a 12-year-old girl with a spontaneously ruptured non-parasitic SC that was successfully treated by minimally invasive laparoscopic dome resection.

## Case presentation

**Fig. 1:**
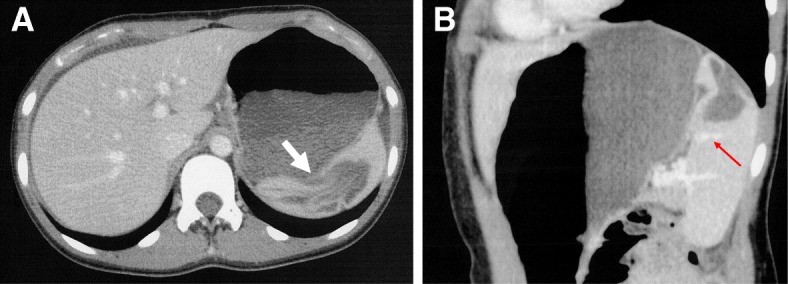
**a** Transverse contrast-enhanced CT images demonstrated a large, collapsed splenic cyst communicating with the peritoneal cavity through the ruptured cyst wall (white arrow), and no neoplastic nodules nor abnormal calcifications were detected. **b** Sagittal contrast-enhanced CT images demonstrated a ruptured cyst with a branch of splenic vessels in the splenic parenchyma near the border of the collapsed cyst at the upper pole of the spleen (red arrow)

A 12-year-old girl presented to a clinic with acute abdominal pain. Her symptoms occurred suddenly, without abdominal trauma, during a school mathematical education class. She was first diagnosed with acute appendicitis by a pediatrician and transferred to our hospital at 5 h after the onset of symptoms. A physical examination showed abdominal distension, with rebound tenderness in the right lower quadrant. Her heart rate was 88 beats/min; blood pressure was 105/66 mmHg. Contrast-enhanced CT demonstrated a collapsed large simple SC (diameter, 7.5 cm) without solid or multifocal component at the upper pole of the spleen (Fig. 1a), and a massive volume of the intraperitoneal fluid was found. A laboratory examination showed that her hemoglobin level was 14.8 g/dl and her leukocyte count was 21700/mm^3^. The serum levels of carbohydrate antigen (CA19-9) and CA125 were 8 U/ml (normal < 37 U/ml) and 12 U/ml (normal < 35 U/ml), respectively. She had no history of abdominal trauma, and cystic Echinococcosis was excluded because she denied any history of traveling abroad. Malignant potential was also excluded, as her tumor makers were negative, and contrast-enhanced CT did not reveal any malignant features, such as heterogeneous, multifocal, irregular nodules [6]. The patient was diagnosed with a spontaneously ruptured non-parasitic SC and peritonitis. As the patient complained of persistent abdominal pain, emergent laparoscopic dome resection of the SC and peritoneal drainage were performed.

## Operative technique

**Fig. 2:**
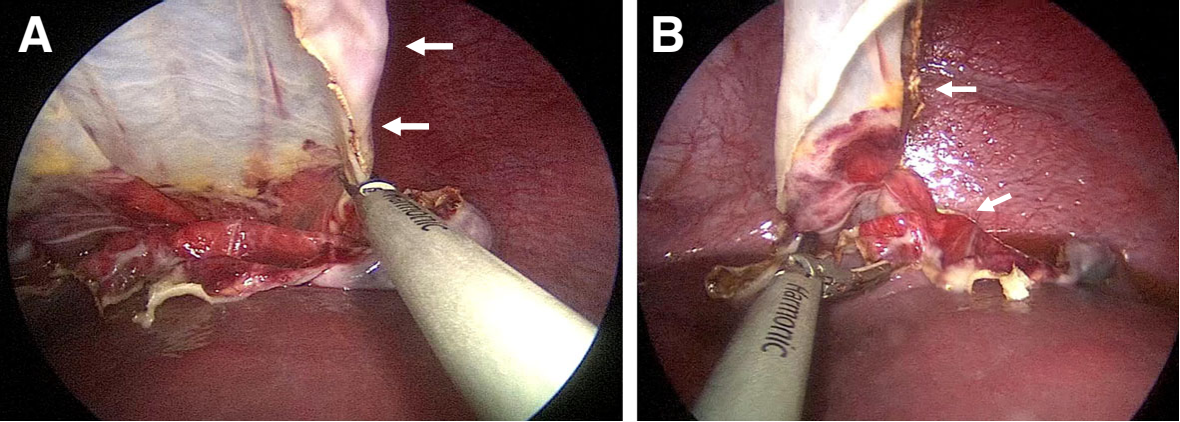
**a**, **b** The protruding part of the cyst wall (white arrow) was entirely resected using a USAD at the border of the splenic parenchyma

**Fig. 3:**
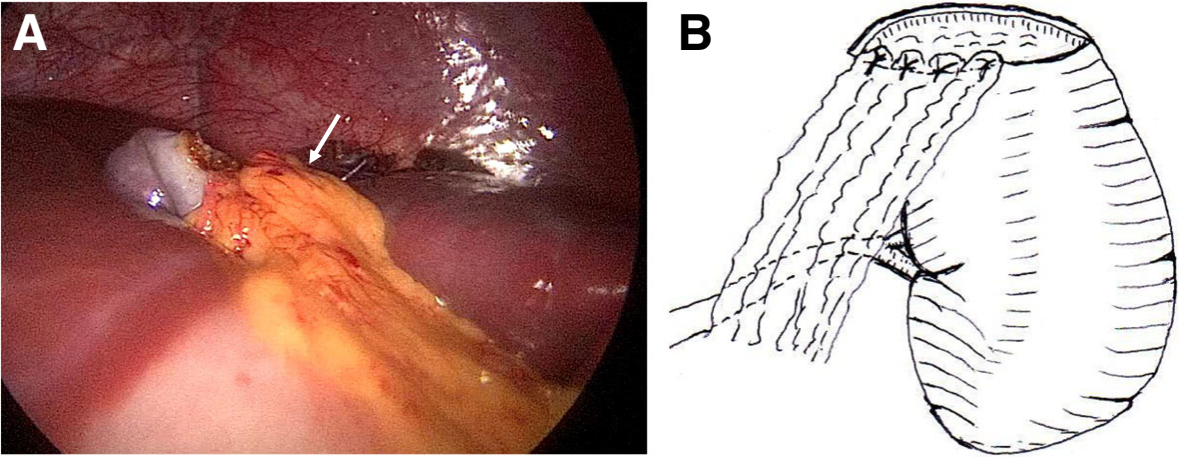
**a b** The greater omentum was sutured to the anterior edge of the splenic parenchyma (arrow) with four interrupted sutures

**Fig. 4:**
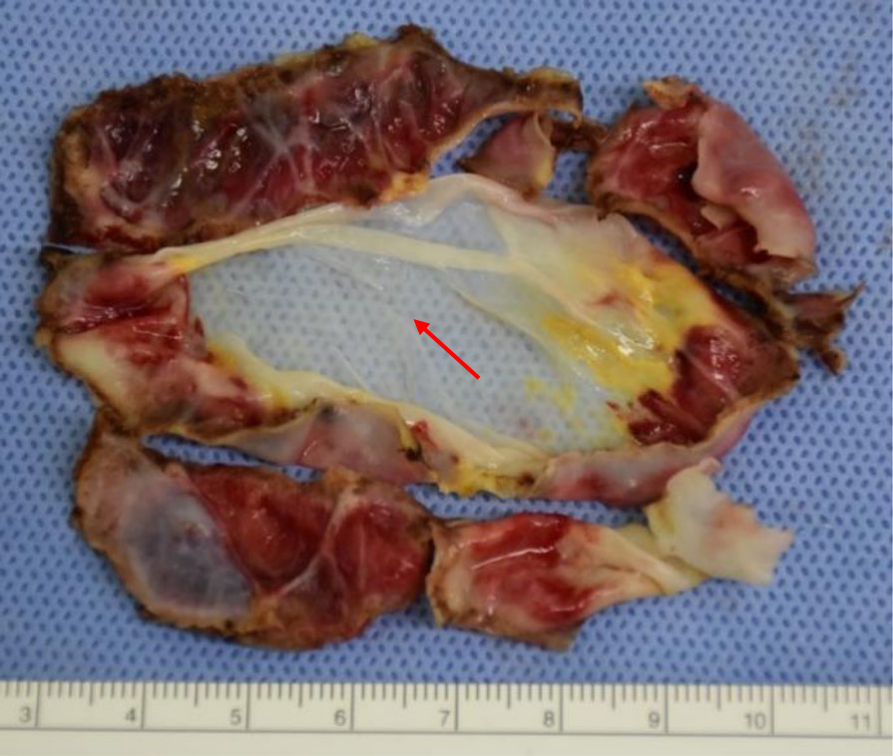
The resected specimen was 7 × 8 cm in diameter, and the ruptured part was a thin wall (red arrow)

After the induction of general anesthesia, the patient was placed in the supine position with her left side elevated. After establishing pneumoperitoneum (8 mmHg, 2 L/min), a 30° laparoscope was inserted through a 12-mm umbilical port, followed by two 5-mm working ports, (one in the left middle abdomen, the other in the right upper quadrant). Laparoscopic abdominal investigation revealed a lot of brownish ascites and a ruptured large cyst in the upper pole of the spleen. The wall of the ruptured cyst showed loose wrinkles. The hilum of the spleen was dissected initially to facilitate the control of bleeding in the event of an emergency, as a branch of the splenic vessels had already been detected in the parenchyma near the border of the collapsed cyst on preoperative enhanced CT (Fig. 1b). After obtaining adequate exposure of the splenic hilum, laparoscopic dome resection of the cyst was performed by excising the entire protruding cystic wall using an ultrasonically activated deice (USAD; Harmonic® scalpel: Ethicon Endosurgery, Inc., Cincinnati, OH, USA), including the thinned part of the splenic parenchyma over the cyst, (Fig. 2a, b). The oozing part of the rim was sprayed with diathermy, and the protruding splenic cyst wall was completely excised. The greater omentum was then sutured to the anterior edge of the splenic parenchyma with intracorporeal knot tying using absorbable threads in order to prevent recurrence of the cyst (Fig. 3a, b), as we speculated that cyst recurrence might develop from the edge of the splenic parenchyma (edge of the remaining cyst) rather than from the remnant cyst floor. After washing the abdominal cavity, a multichannel drain was placed in the left upper quadrant. No complications were observed during the operation. The resected specimen was 7 × 8 cm in diameter (Fig. 4), and a pathological examination of the cyst wall revealed that it was a congenital cyst lined with epithelial cells.

The postoperative course was uneventful at 3 years after surgery, with ultrasound scans showing no evidence of recurrence of the SC.

## Discussion

The management of non-parasitic SCs remains controversial. Small and asymptomatic cysts are often conservatively treated. In contrast, large or symptomatic SCs are usually surgically treated [1, 4]. Despite the risk of severe infection after splenectomy, total splenectomy has been traditionally performed for the ruptured or complicated SCs [2, 5], because some cases have shown life-threatening symptoms [7]. Due to the extremely high risk of infection after splenectomy, several authors have recently performed partial splenectomy or fenestration of the cyst for the treatment of ruptured SCs [3, 8]. Ruptured SCs have only been reported in 9 pediatric patients (including our case) under 15 years of age [3, 8–14] (Table 1). Most patients were of pubertal age, and the most common symptom was acute abdominal pain. The rupture occurred spontaneously in six patients (cases A, C, D, E, F, I) and was triggered by trauma in three patients (cases B, G, H). Total splenectomy was performed in six patients (cases A, B, C, D, E, F), and partial splenectomy was performed in one patient (case H). Laparoscopic surgery was performed in two patients (cases G, I); one patient underwent delayed laparoscopic fenestration at 4 weeks after the initial admission (case G), while our other present patient (case I), underwent emergent laparoscopic dome resection with preservation of the splenic parenchyma. All cases involved cysts with an epidermoid or epithelial histology. Although laparoscopic techniques for managing splenic cysts, including fenestration, decapsulation, and marsupilation with or without omental packing, are simple and minimally invasive procedures, it has been reported to be associated with a high rate of cyst recurrence [15–17]. Recurrence of the cyst has been frequently observed at a median of 6–12 months after initial surgery in cases with laparoscopic partial cyst wall resection [15, 16]. Some authors have suggested that total splenectomy is safe and necessary in cases such as those of a large cyst (> 5 cm), with dense vascular adhesions to the surrounding tissues and structures, as simple decapsulation results in repeated recurrence [17]. In our case, there were no adhesions to the surrounding structures, and the protruding part of the cystic wall was able to be completely resected with an adequate margin for the splenic parenchyma using a USAD with only slight oozing. In addition, suturing the greater omentum to the edge of the splenic parenchyma might have helped to prevent the regrowth of the cystic wall, as no recurrence of the SC was observed at 3 years after the operation.

## Conclusions

Laparoscopic complete resection of the protruding part of the wall of SC using a USAD and omental suturing to the edge of the splenic parenchyma may be a feasible and effective procedure for managing ruptured large non-parasitic SCs in pediatric patients.

## Data Availability

All datasets supporting the conclusions of this article are included within the article.

## Abbreviations

SC: Splenic cyst
CT: Computed tomography
USAD: Ultrasonically activated device
CA19-9: Carbohydrate antigen 19-9
CA125: Cancer antigen 125
Inc.: Incorporated
HO: Ohio
USA: United States of America
